# Assessing Biodiversity in Agricultural Landscapes Through Drone‐Based Environmental DNA Sampling

**DOI:** 10.1002/ece3.74147

**Published:** 2026-08-11

**Authors:** Camille Albouy, Louise Humbert, Kerstin Niedermeier, Marc Chautem, Fabian Fopp, Martina Luthi, Sarah Thurnheer, Virginie Marques, Steffen Kirchgeorg, Christian Geckeler, Ali Nasrallah, Stefano Mintchev, Loïc Pellissier

**Affiliations:** ^1^ Ecosystems and Landscape Evolution, Department of Environmental Systems Science ETH Zürich Zürich Switzerland; ^2^ Swiss Federal Institute for Forest Snow and Landscape Research WSL Birmensdorf Switzerland; ^3^ Axa Climate Paris France; ^4^ Environmental Robotics Laboratory, Department of Environmental Systems Science ETH Zürich Zürich Switzerland

## Abstract

As agricultural intensification expands globally, there is an increasing concern about the impact of food production on global biodiversity. Biodiversity decline is problematic as species provide a wealth of benefits, including pollination, soil fertility, and protection against pests, within agrosystems. Many countries, especially across Europe, have implemented incentives for farmers to introduce biodiversity‐friendly land management practices, from building hedgerows to planting pollinator fields. Quantifying the impacts of these measures on biodiversity at the farm scale is technically challenging. Here, we developed a method involving the collection of environmental DNA (eDNA) samples with drones from crops, demonstrated in a case study on rapeseed fields under three management types: conventional, biological, and IP Suisse. We analyzed swabbed material through metabarcoding of a 16S amplicon to detect the composition of hexapod in the field. After cleaning and taxonomic assignment, we obtained a total of 75 taxa assigned to 19 families, 23 genera, and 33 species. We found that the variance in recovered diversity was significantly higher for replicates between fields than for replicates within a field, suggesting that eDNA swabbing replicates provided consistent local results. We did not detect significant differences between treatments, possibly because of a landscape effect which causes spillover of species from neighboring seminatural habitats. Our results provide a direction for developing a toolbox for biodiversity measurements in agricultural fields, highlighting the potential for expanding these methodologies to suit the needs of scientists, farmers, and other stakeholders in understanding and fostering farm‐scale biodiversity.

## Graphical abstract

We developed a drone‐based environmental DNA (eDNA) swabbing method to assess hexapod diversity in agricultural fields under three management types (conventional, organic, and IP‐Suisse), detecting 75 taxa across 19 families. eDNA replicates showed consistent within‐field results, though no significant differences in diversity were found between management types. This proof‐of‐concept demonstrates the potential of drone‐assisted eDNA swabbing as a scalable, noninvasive tool for farm‐scale biodiversity monitoring.

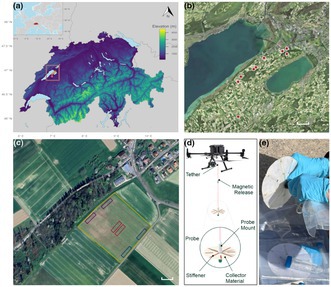

## 1 Introduction

Among the greatest concerns in ecology today is the alarming global decline in biodiversity (Pimm et al. 2014; Cardinale et al. 2012; Newbold et al. 2015). This decline, particularly evident in insects, is strongly linked to agricultural practices, where pesticide use and land management have significantly contributed to biodiversity loss (e.g., Uhler et al. 2021; Raven and Wagner 2021). As global demand for agricultural products continues to rise with population growth, agricultural intensification, and its associated threats to biodiversity, has also increased (Uchida and Ushimaru 2014). Agricultural landscapes, which account for one of the largest land uses worldwide, play a critical role in shaping species distributions and their habitats (Duelli and Obrist 2003a; Billeter et al. 2008; Clergue et al. 2009). The shift from diverse farming practices to monocultures, driven by economic pressures to maximize yields, has significantly reduced habitat availability and quality for biodiversity. Consequently, intensive agricultural management has led to widespread species loss and disruptions to essential ecosystem services such as pollination, soil fertility and pest control. It remains challenging to quantitatively evaluate the impact of agricultural practices on biodiversity at the field scale (Bullock et al. 2021; Dominati et al. 2021; Letourneau and Bothwell 2008), in part due to the absence of accessible methodologies. To address this gap, there is an urgent need to develop and implement scalable, field‐based biodiversity monitoring methods that align with global conservation goals while supporting agricultural productivity (Reidsma et al. 2006).

A variety of agricultural practices have been developed to promote biodiversity, reflecting the diversity of crops and farming systems worldwide (e.g., Tamburini et al. 2020; Thrupp 2004). Management practices on farmland are extremely diverse and can vary along axes of mechanization (e.g., Orpet et al. 2020), chemical use (Panico et al. 2022; Benbrook et al. 2021), the use of compensation area (Ravetto Enri et al. 2020), and frequency of treatments (Nicholson and Williams 2021). For example, while the use of pesticide, distinguishes conventional and organic farming, there are large variations in practices within these classes including usage of heavy machinery, which can be more intense in organic farming to compensate for prohibited herbicide use (Orpet et al. 2020). In the extreme cases, some organic farming programs are fully dedicated to promoting biodiversity through crop rotation, habitat protection, and the use of minimal chemicals. Studies comparing these approaches indicate that organic farming increases species richness by approximately 30% compared to conventional farming (Tuck et al. 2014; Hole et al. 2005; Reidsma et al. 2006), which highlights the potential for organic practices to mitigate biodiversity loss. However, in most cases, it is difficult to define a clear link between agricultural practices and biodiversity, and thus, the only approach is to make empirical biodiversity measurement on the farm (Elmiger et al. 2023). The agricultural transition movement emphasizes a shift toward more sustainable production methods, including reduced tillage, crop rotation diversification, cover crops, temporary grasslands, improved nitrogen management, compost use, pesticide reduction, agroforestry, and the establishment of hedgerows (Tscharntke et al. 2021). However, the lack of regular and standardized biodiversity monitoring has hindered the effective implementation and evaluation of impacts of more ecological management practice.

Biodiversity monitoring on farms often focuses on key taxa such as pollinators and plants (Rodríguez‐Gasol et al. 2020), but so far biodiversity quantification is not globally standardized due to regional differences in flora, fauna, crop types, and the lack of universally accepted tools. Commonly monitored groups include vascular plants, pollinators (such as bees, butterflies, and hoverflies), birds, and soil organisms like earthworms and beetles, as these taxa are often associated with ecosystem health and agricultural productivity (Duelli and Obrist 2003b; Tscharntke et al. 2005). In Europe, biodiversity monitoring is frequently tied to farmer incentives, with biodiversity‐friendly practices linked to monetary compensation (Magda et al. 2015; Elmiger et al. 2023). Monitoring schemes are developed nationally or subnationally and often rely on presence/absence data of target species organisms as proxies for overall biodiversity (Tasser et al. 2019). Common European biodiversity indicators include specific vascular plant species, orchard tree size, bird nest presence, dung near water sources, ground cover, and structural features like hedgerows and dry walls (Elmiger et al. 2023). However, these indicators are region‐specific and lack universal applicability, making cross‐regional farm comparisons as well as generalization challenging. In addition, these traditional approaches are highly labor‐intensive, requiring expert taxonomists for accurate species identification, which can be particularly challenging for morphologically similar or cryptic taxa, such as certain insect species or fungi. These methods are further constrained by their reliance on in‐field observations, which often overlook cryptic, nocturnal, or less conspicuous species. Moreover, traditional methods are limited by spatial and temporal coverage, as monitoring large agricultural areas over extended periods is both costly and time‐consuming (Billeter et al. 2008). In intensively managed or highly diverse landscapes, the high sampling effort required to detect all taxa present often makes comprehensive biodiversity assessments impractical. Consequently, these limitations restrict our ability to obtain robust, long‐term biodiversity data in agricultural systems, highlighting the need for more efficient and scalable approaches.

Environmental DNA (eDNA) is increasingly transforming biodiversity monitoring by providing a noninvasive, comprehensive method to detect species across diverse ecosystems (Beng and Corlett 2020; Hassan et al. 2022; Drummond et al. 2015). eDNA consists of DNA traces shed by organisms into their environment, which can be collected from sources such as water (Deiner et al. 2016; Harper et al. 2019; Lyet et al. 2021), air (Lynggaard et al. 2022; Roger et al. 2022), surfaces (Valentin et al. 2020), and soil (Allen et al. 2023; Todd et al. 2020; Kirse et al. 2021). The standard eDNA workflow includes sample collection, DNA extraction, sequencing, and bioinformatics analysis to identify species, offering insights into species presence, species richness, and community composition. Despite its growing use in conservation, eDNA applications in agriculture remain limited, comprising only 4% of published eDNA studies screened by Kestel et al. (2022) compared to 48% addressing broader ecological and taxonomic questions. However, the potential for eDNA in agricultural biodiversity monitoring is vast, including applications for pest and pathogen detection, identifying indicator species, and assessing soil health (Kestel et al. 2022). By linking eDNA analysis with farming practices, agriculture can benefit from a more dynamic understanding of biodiversity, enabling more informed management decisions.

Robots are increasingly being integrated into agricultural practices, revolutionizing tasks such as spraying, harvesting, and planting with greater precision and efficiency (Vougioukas 2019). These advancements in robotic technologies are helping address challenges associated with managing large agricultural surfaces manually, which are time‐consuming and labor‐intensive. Beyond crop management, robots hold significant potential for biodiversity monitoring in agricultural landscapes. Equipped with sensors, cameras, and automated sampling tools, drones can help systematically collect eDNA samples in complex environments such as tree branches (Aucone et al. 2023) or assess diversity across expansive grassland areas (Koubínová et al. 2025) or tropical rainforests (Kirchgeorg et al. 2024; Geckeler et al. 2025). By automating biodiversity monitoring, robots could overcome the logistical limitations of manual sampling, enabling more frequent, spatially extensive, cost‐effective, and repeatable data collection. To reach this goal, it is necessary to assess the eDNA signal recovered by drones over crop fields, which has not yet been evaluated. The integration of robotics with biodiversity monitoring offers promising opportunities for scaling up sustainable agricultural practices while ensuring the preservation of ecological health.

In this study, we investigate the use of eDNA metabarcoding collected from swabbing crops using drones to compare hexapod diversity in three different agricultural managements, from conventional to organic farming. In Switzerland, there has been a significant modification in arthropod diversity (Martínez‐Núñez et al. 2024; Neff et al. 2022) and abundance (Artmann‐Graf and Korner 2024; Neff et al. 2025), which has far‐reaching consequences for higher trophic levels, including many bird species (Planillo et al. 2021). They occupy a wide range of functional roles, from pollinators and decomposers to predators and herbivores, and are present in all vegetation types, including cropland (Yang and Gratton 2014). As such, monitoring hexapod diversity provides insights into the overall health and functioning of an ecosystem. Another advantage of focusing on hexapod is that they live directly on the plants, thus expected to leave DNA traces on vegetation through contact, such as through feces, secretions, and bodily fragments, which can be effectively collected using swabbing methods. Here, we study the potential of eDNA‐based monitoring in agricultural systems, from conventional, organic, and IP‐Suisse crops. We asked the following questions:

1. Can we recover hexapod eDNA from crops using swabbing drones, and what are the main taxonomic groups recovered?
2. Do we observe a lower variance in diversity within‐field than between‐field, suggesting that the method allows the level of insect diversity to be consistently measured in a specific field?
3. Is there a difference in hexapod diversity and composition between the three agricultural treatments: conventional, biological, and IP‐Suisse?

## 2 Materials and Methods

### 2.1 Study Area and Crop Selection

**FIGURE 1:**
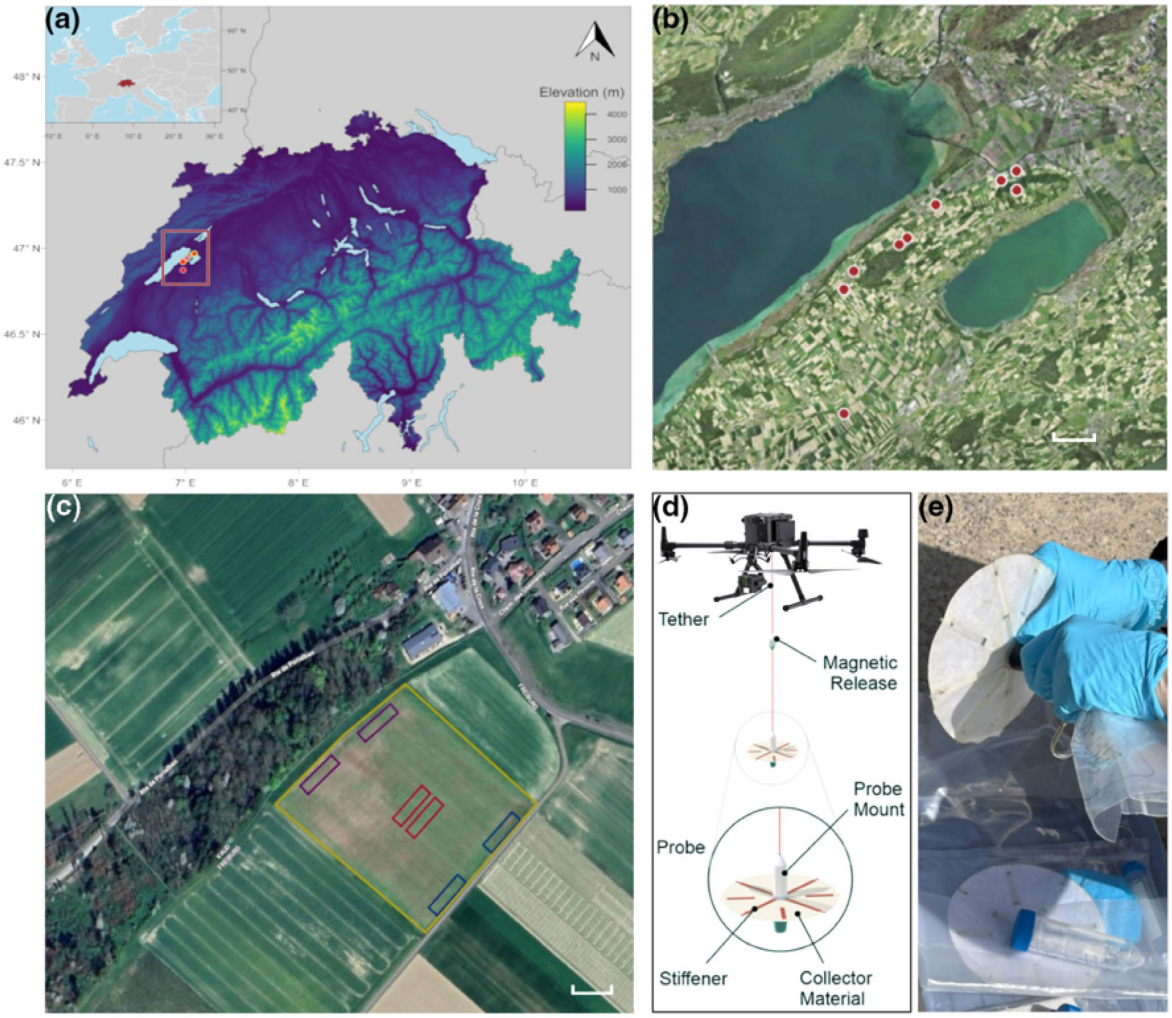
Map of the sampling locations across Switzerland (a), with a zoom on the agricultural region of La Broye (canton of Vaud, VD) (b) and an example of the six sampling locations is represented, with rectangles indicating the area covered by the drone over one of the selected fields (c). The illustration (d) shows the drone sampling mechanism, particularly the design of the probe for sampling the field (adapted from Koubínová et al. 2025) and the photo (e) shows one probe in its sterile bag, with another mounted on the drone ready for sampling. The backgrounds of panels b and c were extracted from Google Maps.

Fieldwork was conducted in Switzerland (Figure 1a), in the La Broye region (canton of Vaud, VD). We selected the fields from a delimited area located on the Swiss Plateau, with major crop cultivation (Figure 1b). We focused on rapeseed ( *Brassica napus* ), which represents the third largest crop area in Switzerland, with a total of 9% of agricultural surfaces (FSO 2025, agristat). Rapeseed grows up to 1.70 m during the summer with major flowering events, potentially providing habitats and resources to numerous hexapod species. We sampled this area at the end of May (26th, 28th) and at the beginning of June (5th, 6th) 2024. At that time, the flowering stage of rapeseed was over, the plants were already podding. We obtained authorization from all participating farmers, who granted access to their rapeseed fields for the experimental work. We selected a total of nine rapeseed fields: three conventionally managed, three organically managed as well as three fields with IP‐Suisse (Swiss association of Integrated Producing farmers) management. For the year 2024, rapeseed cultivation under IP‐Suisse management involved farming without insecticides nor fungicides but solely with herbicides and alternatives to chemicals. From these fields, we initially collected nine samples from conventional, twelve from IP‐Suisse, and nine from organic fields. Due to conservation issues affecting some samples (e.g., leakage and then insufficient buffer volume for DNA extraction), the final dataset comprised eight samples from organic fields, eight from conventional fields, and eleven from IP‐Suisse fields.

### 2.2 Sample Collection

To sample the differently managed fields, we used a DJI Matrice M300 drone equipped with a specialized eDNA probe (Figure 1d). The probe is attached to the drone via a four‐meter tether attached with Velcro, with a central magnetic release mechanism that allows easy attachment and detachment while preventing crashes if the probe becomes caught in vegetation. The eDNA is collected using a moisturized circular swab, reinforced with four fiberglass stiffeners to maintain its shape. The probe collects samples by swabbing vegetation while being dragged horizontally or during vertical movements through the crops (Koubínová et al. 2025). For each field, we then defined the areas for the six replicates, two of them being close to the center (see Figure 1c). The other four areas were located on two opposite sites close to the edge of the field, while respecting a 15 m buffer from the edge of the field to avoid edge effects (Holland and Fahrig 2000). We flew the drone for 5 min for each replicate and above a 50 m x 15 m area, following a pattern along the edges of the rectangle with progressively decreasing side lengths toward the center. The areas designed for the replicates did not overlap spatially (Figure 1c). We used sterile sampling kits prepared beforehand in a clean lab, consisting of one circular swab (15 cm diameter) with stiffeners, a pair of sterile gloves and a 15 mL tube filled with 10 mL of ddH2O and a 50 mL tube filled with 10 mL of Longmire buffer (Longmire et al. 1997). Sampling was done under stable weather conditions with less than 8 mm of precipitation on the day before the sampling, and no precipitation during the sampling. After defining the sampling area, we moistened the swab with the ddH2O water and attached the probe to the drone (Figure 1d, Kirchgeorg et al. 2024). The drone then flew over the designated area of the rapeseed field, towing the dampened swab to sample eDNA. We removed the circular swab from the drone by using a pair of sterile gloves. The filter was put directly into the 50 mL tube containing Longmire buffer and kept in an opaque box at room temperature (20°C–24°C) until extraction. Between each sample, we decontaminated the probe and all utensils with 10% bleach and rinsed with ddH_2_O to prevent cross‐contamination. As Longmire buffer is well suited for long‐term DNA preservation, we stored the samples for 25 to 36 days prior to extraction without compromising DNA integrity.

### 2.3 Swabs eDNA Extractions and Amplification

DNA collected from surface swabs was extracted using a protocol based on Pont et al. (2018). Before entering the clean laboratory dedicated to eDNA sample processing, we decontaminated the exterior of Falcon tubes containing field‐collected swabs with 10% bleach, and the tubes were subsequently vortexed. In the clean lab, we removed the swabs from the tubes using sterile tweezers and placed them into 50 mL syringes. Then we compressed the swabs to collect any buffer solution absorbed during storage, which, together with the remaining buffer from field collection, was used for DNA extraction. DNA was precipitated using an ethanol precipitation and ATL Buffer (from Qiagen DNEasy Blood and Tissue Kit) and purified and eluted using an adapted protocol of the NucleoSpin Soil Kit from Machery‐Nagel (Macherey‐Nagel GmbH & Co., Germany). Once the DNA was extracted, we stored the samples at −20°C. Before proceeding with library preparation, we first performed a quantitative PCR (qPCR) using the same primers and conditions as the standard PCR (see below), to assess the presence of potential inhibitors in our DNA extracts that could interfere with downstream applications. This quality‐control step was crucial for ensuring reliable and consistent results throughout library preparation. To evaluate potential inhibition, each sample was analyzed as an undiluted extract as well as at 1:10 and 1:100 dilutions. No evidence of PCR inhibition was detected in any sample. We then proceeded with library preparation using a two‐step PCR protocol. To amplify insect DNA, we used the 16S hexapod‐specific primers; INS‐16S‐Forward: 5′‐GGACGAGAAGACCCTWTAGA‐3′ and INS‐16S‐Reverse: 5′‐ATCCAACATCGAGGTCGCAA‐3′. The PCRs was performed in a mix containing AmpliTaq, BSA and DMSO with the following thermocycling conditions: a first denaturation step at 95°C for 10 min followed by 40 cycles of a second denaturation (95°C, 30s) with an annealing step (55°C, 30s) and the extension step (72°C, 14 s) and a final extension at 72°C for 5 min. We performed 8 PCR replicates for each sample, which we pooled before sending them for sequencing at Microsynth. The samples were sequenced through Illumina NextGen sequencing on an Illumina NovaSeq, 2 × 250 bp, with a target of 200 k reads per sample, with a demultiplexing and trimming of the adaptor residuals.

### 2.4 Bioinformatics and Taxonomic Assignment

The bioinformatic analyses were performed using the dada2 pipeline (Callahan et al. 2016) to generate Amplicon Sequence Variants (ASVs). We performed the taxonomic assignment of ASVs against the MIDORI2 (GB265) database, with species‐level matches accepted only at 100% sequence identity. Following taxonomic identification, all nonhexapod ASVs were removed. Chimeric sequences were checked and removed as part of the dada2 pipeline. We also performed a second independent taxonomic assignment of consensus sequences using blastn (v2.15.0+; Camacho et al. 2009) against the MIDORI2 16S reference database (vGB265). We applied an e‐value threshold of 1e‐3 and required a minimum of 80% identity and coverage, retaining the top 20 hits for each consensus sequence when available. Species‐level assignments were accepted for sequences with ≥ 100% identity, genus‐level with ≥ 98% and family‐level with ≥ 96%. In cases where multiple hits shared the highest bit‐score, we retained only the common taxonomic levels among these top‐ranked hits. To generate a consolidated taxonomic data set, we merged the dada2 and blast outputs by sequence identity, retaining the most complete taxonomic assignment for each amplicon sequence variant (ASV). When both pipelines provided assignments, we selected the taxonomy with the higher level of completeness (i.e., the greatest number of resolved ranks from kingdom to species). The resulting data set was then processed at multiple taxonomic levels. For species‐level analyses, ASVs identified to species were aggregated by summing read counts across samples. The same procedure was applied for genus‐ and family‐level assignments, ensuring that each taxonomic rank was represented by the most complete and nonredundant information available. Species, genus, and family tables were then merged into a unified taxonomic data set. To reduce contamination, reads detected in blank controls were subtracted from samples, and we retained taxa belonging to Insecta or Collembola. Finally, rare taxa with fewer than 10 reads per sample were excluded from downstream analyses.

With the remaining ASVs, we performed a taxa accumulation analysis to assess the number of samples required to capture the entire hexapod diversity across the three considered management types: conventional, biological and IP‐Suisse. We used the specaccum and fitspecaccum functions from the R package vegan (Oksanen et al. 2025), which calculate the expected taxa accumulation curve using a sample‐based rarefaction method and then fit nonlinear accumulation models. We tested three different models (Lomolino, asymptotic and logistic) and identified the best fit by selecting the model with the lowest Akaike Information Criterion (AIC) for each management type. Based on the AIC, we then fitted the best model to the data and estimated the asymptote value for each type of management.

### 2.5 Diversity Variation Within and Between Field

To assess and compare the hexapod diversity among different fields with different management types, we computed diversity metrics, specifically the taxa richness and the hill number (q1) as a measure of evenness (Chao et al. 2014) for each sample. Considering both species richness and evenness after testing for the variance homogeneity (Levene's test), we compared the variance of diversity within and between fields using a one‐way type II ANOVA to account for the unbalanced number of replicates and to assess whether the within‐field variance significantly differed from the between‐field variance.

To compare diversity levels across management types and after confirming no overdispersion (*d* = 0.88, *z* = −0.60, *p*‐value = 0.73), we fitted a Poisson general linear model to explain taxa richness as a function of management types and fields. We checked the homogeneity of variance of the explained variable in each management type and the model residuals for normality by applying both a Levene and a Shapiro test (Royston 1982). For the Poisson general linear model, we calculated the estimated marginal means (EMMs) on the log‐transformed scale for each management type, and we used the Tukey method for multiple comparisons to test for pairwise differences between management types.

As evenness is a continuous variable, we first fitted a linear model to explain evenness as a function of management types and fields. The model residuals were normally distributed, but evenness varied significantly among management types (Levene's test, *F* = 6.85, *p* = 0.0044), indicating unequal variance. Therefore, we subsequently tested the influence of management types and Field on evenness (q1) using a linear mixed‐effects model. Management types were included as a fixed effect, while Field was included as a random intercept to account for the nested structure of the data. The significance of fixed effects was assessed with an ANOVA Type III Wald *χ* ^2^ test. We tested whether taxa richness and evenness differ across management types.

### 2.6 Composition Difference

For assessing β‐diversity, we created a presence–absence matrix based on the taxa detected, and we calculated the pairwise Jaccard dissimilarity along with its two additive components, the taxa turnover (βjtu) and the nestedness (βjnes) between sites (Anderson et al. 2011; βjac) by using the function “beta.pair” of the R package ‘Betapart’ (Baselga and Orme 2012). We then performed a Permutational Multivariate Analysis of Variance (PERMANOVA) on both the βjac and βjtu to assess the effects of management type, sampled field and time (number of days between samples) on differences in hexapod composition. To visualize and analyze the compositional differences among the management types, we performed a Principal Coordinates Analysis (PCoA) based on both βjac dissimilarity matrices. This ordination method allowed the identification of patterns in hexapod community composition and assessed how distinct or similar the communities were across different management types. The results were interpreted in conjunction with the statistical significance from PERMANOVA, providing a comprehensive understanding of the hexapod community structure in relation to the management types swabbed.

## 3 Results

### 3.1 Number of Reads and Species Diversity

**FIGURE 2:**
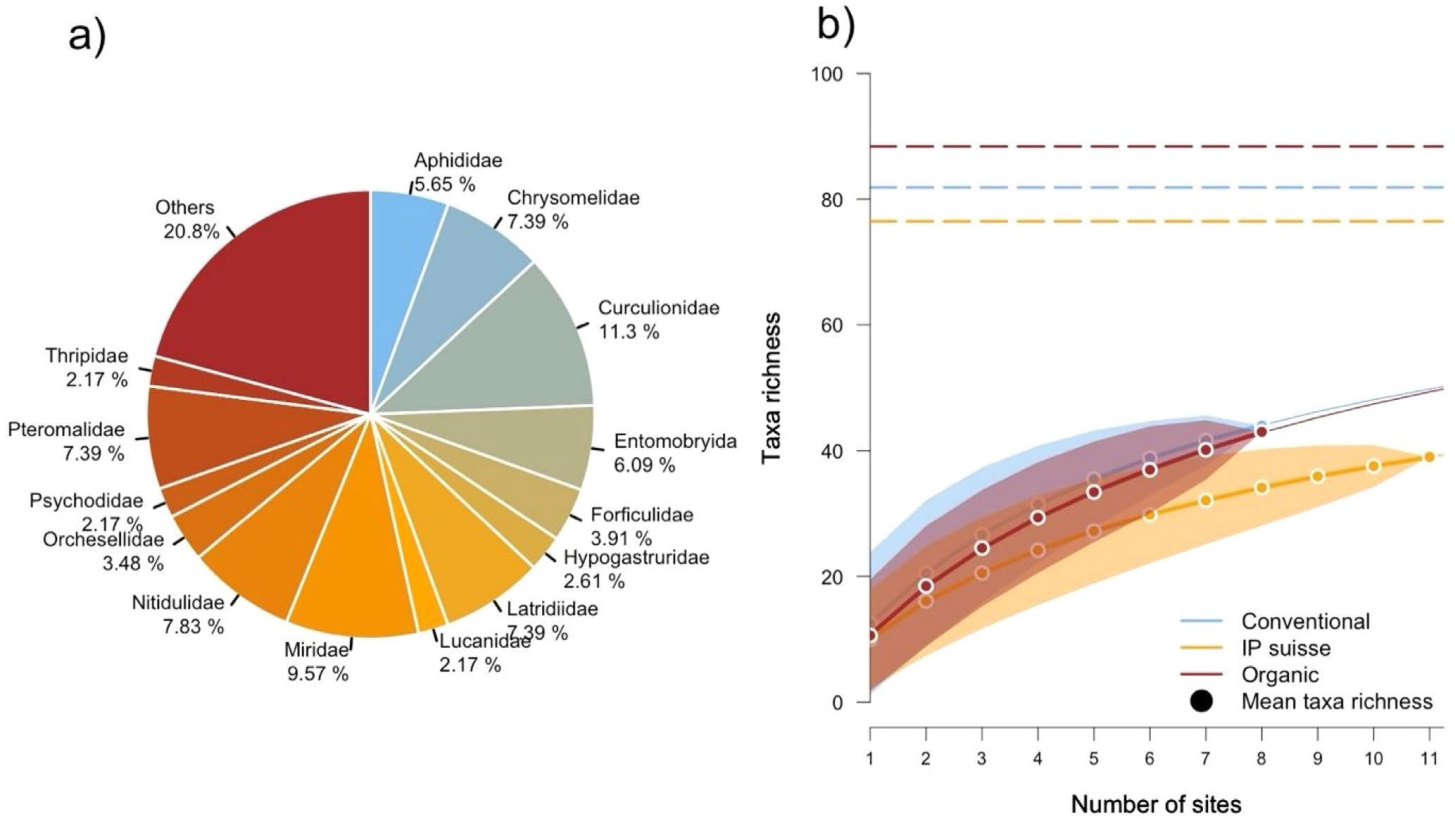
(a) Distribution of the hexapod families detected from crops expressed as relative proportions of the total community. (b) Species accumulation curve for the three management types, conventional, IP Suisse and organic. The solid circles indicate the mean taxa richness observed for each management type at the corresponding number of sites sampled. The colored envelope represents the standard deviation around the mean taxa richness.

A total of 3,150,657 reads were obtained from the sequencing of the 9 rapeseed fields and after trimming and excluding nonhexapod taxa. When comparing the total read counts per collected sample, the distribution was even between management types. Organic fields have a mean of 108,435 reads per sample (+/− 5957 SD), IP‐Suisse fields 108,177 reads per sample (+/− 8672), and conventional fields 109,646 reads per sample (+/− 3771). In total, we detected 536 hexapod ASVs, assigned to 78 families, 118 genera and 69 species. After cleaning and taxonomic assignment, we obtained a total of 755,207 reads representing 75 taxa assigned to 19 families, 23 genera and 33 species. The most represented taxonomic orders are Coleoptera (beetles) with the Curculionidae and Chrysomelidae families representing 11.3% and 7.39% of the total reads, respectively (Figure 2a), followed by Hemiptera (true bugs) and Diptera (flies). Of the 33 species identified, the plant bug (Lygus_pratensis/Lygus_wagneri) with 28,3210 reads (35% of total species reads) is the most represented, followed by the leaf beetle (Oulema rufocyanea) with 71,624 reads (9% of total species reads) and the two‐spotted grass bug ( *Stenotus binotatus* ) with 41,712 reads (5% of total species reads) (Table 1). The remaining species showed a considerably lower number of reads. The species accumulation curves did not reach a plateau, showing that our sampling of these fields did not fully represent the regional diversity of the studied agro‐ecosystem (Figure 2b).

**TABLE 1:** Taxa detected in different management types, conventional, organic and IP‐Suisse, indicating their number of occurrences and the number of reads obtained per taxon in each management type.

Management types	Class	Order	Family	Genus	Taxa	Nb occ	Nb read
Conventional	Collembola	Entomobryomorpha	Entomobryidae	NA	Entomobryidae	4	2486
		Poduromorpha	Hypogastruridae	NA	Hypogastruridae	2	12,799
	Insecta	Coleoptera	Anthicidae	Anthicus	*Anthicus antherinus*	2	543
			Carabidae	Loricera	Loricera	1	233
			Chrysomelidae	NA	Chrysomelidae	2	182
				Oulema	*Oulema rufocyanea*	3	71,490
				Phyllotreta	Phyllotreta	2	48
				Psylliodes	Psylliodes	2	179
				Psylliodes	*Psylliodes chrysocephalus*	2	180
			Curculionidae	NA	Curculionidae	5	1757
				Ceutorhynchus	Ceutorhynchus	3	20,625
					*Ceutorhynchus pallidactylus*	8	6493
				Rhynchaenus	*Rhynchaenus fagi*	3	281
				Sitona	Sitona	2	186
			Elateridae	Selatosomus	Selatosomus	1	189
			Erirhinidae	Dorytomus	*Dorytomus longimanus*	1	327
			Latridiidae	Cortinicara	Cortinicara	5	257
			Lucanidae	Dorcus	*Dorcus parallelipipedus*	2	43
			Melyridae	NA	Melyridae	2	129
			Nitidulidae	NA	Nitidulidae	3	1200
			Staphylinidae	NA	Staphylinidae	1	17,286
				Tachyporus	*Tachyporus hypnorum*	1	49
		Dermaptera	Forficulidae	Forficula	*Forficula auricularia*	3	612
		Diptera	Limoniidae	Dicranomyia	Dicranomyia	1	9395
			Psychodidae	NA	Psychodidae	2	4657
			Stratiomyidae	NA	Stratiomyidae	1	32
		Ephemeroptera	Caenidae	Caenis	*Caenis horaria*	1	13
			Ephemerellidae	Serratella	Serratella	1	11
			Heptageniidae	Rhithrogena	*Rhithrogena semicolorata*	1	112
		Hemiptera	Acanthosomatidae	Acanthosoma	*Acanthosoma haemorrhoidale*	1	932
			Aphididae	Brevicoryne	*Brevicoryne brassicae*	4	235
				Myzus	Myzus	2	91
			Aphrophoridae	Philaenus	Philaenus	1	11
			Miridae	NA	Miridae	1	1200
				Lygus	Lygus	3	3633
					*Lygus pratensis/Lygus wagneri*	6	178,686
		Hymenoptera	Formicidae	Lasius	*Lasius niger*	2	175
			Pteromalidae	NA	Pteromalidae	7	2649
		Orthoptera	Tettigoniidae	Tettigonia	*Tettigonia viridissima*	1	175
		Psocoptera	Caeciliusidae	NA	Caeciliusidae	1	196
			Liposcelididae	Liposcelis	*Liposcelis decolor*	1	43
			Psocidae	Loensia	*Loensia variegata*	1	19
			Psyllipsocidae	Dorypteryx	Dorypteryx	1	233
		Thysanoptera	Thripidae	Thrips	*Thrips tabaci*	1	17
IP Suisse	Collembola	Entomobryomorpha	Entomobryidae	NA	Entomobryidae	4	2727
			Orchesellidae	NA	Orchesellidae	2	1759
				Orchesella	*Orchesella villosa*	2	90
		Poduromorpha	Hypogastruridae	NA	Hypogastruridae	3	221
	Insecta	Coleoptera	Chrysomelidae	NA	Chrysomelidae	1	125
				Oulema	*Oulema melanopus*	1	111
					*Oulema rufocyanea*	2	97
				Psylliodes	Psylliodes	2	110
					*Psylliodes chrysocephalus*	3	329
			Coccinellidae	Coccinella	*Coccinella septempunctata*	1	112
			Curculionidae	NA	Curculionidae	6	1237
				Ceutorhynchus	Ceutorhynchus	9	1987
					*Ceutorhynchus pallidactylus*	5	1225
				Rhynchaenus	*Rhynchaenus fagi*	4	335
				Sitona	*Sitona lineatus*	1	225
			Latridiidae	Cortinicara	Cortinicara	8	96,865
			Lucanidae	Dorcus	*Dorcus parallelipipedus*	1	71
			Nitidulidae	NA	Nitidulidae	10	2571
				Brassicogethes	Brassicogethes	4	715
			Ptinidae	Stegobium	*Stegobium paniceum*	1	78
			Staphylinidae	Tachyporus	*Tachyporus hypnorum*	1	476
			Tenebrionidae	Tribolium	*Tribolium castaneum*	2	618
		Dermaptera	Forficulidae	Forficula	*Forficula auricularia*	2	5095
		Diptera	Psychodidae	NA	Psychodidae	3	60
			Sciaridae	Scatopsciara	Scatopsciara	2	1427
		Ephemeroptera	Ephemerellidae	Serratella	Serratella	1	20
		Hemiptera	Anthocoridae	Orius	Orius	1	226
			Aphididae	Brevicoryne	*Brevicoryne brassicae*	1	936
				Myzus	*Myzus persicae*	2	280
				Sitobion	*Sitobion avenae*	1	143
			Miridae	Leptopterna	*Leptopterna dolabrata*	1	1480
				Lygus	Lygus	3	776
					*Lygus pratensis/Lygus wagneri*	8	43,879
		Hymenoptera	Formicidae	Lasius	*Lasius niger*	1	11
			Pteromalidae	NA	Pteromalidae	6	10,795
		Orthoptera	Acrididae	Chorthippus	Chorthippus	1	424
			Acrididae	NA	Acrididae	1	136
		Psocoptera	Lachesillidae	NA	Lachesillidae	1	19
		Thysanoptera	Thripidae	Thrips	*Thrips tabaci*	2	69
Organic	Collembola	Entomobryomorpha	Entomobryidae	NA	Entomobryidae	4	1167
				Willowsia	Willowsia	1	22
					*Willowsia nigromaculata*	1	1838
			Orchesellidae	NA	Orchesellidae	3	103
		Poduromorpha	Hypogastruridae	NA	Hypogastruridae	1	20
	Insecta	Coleoptera	Chrysomelidae	Oulema	*Oulema rufocyanea*	2	37
				Phyllotreta	Phyllotreta	1	34
				Psylliodes	Psylliodes	2	647
				Psylliodes	*Psylliodes chrysocephalus*	4	325
			Chrysomelidae	NA	Chrysomelidae	1	47
			Coccinellidae	Harmonia	Harmonia	1	303
			Curculionidae	NA	Curculionidae	3	17,471
				Ceutorhynchus	Ceutorhynchus	5	4002
					*Ceutorhynchus pallidactylus*	6	4456
				Rhynchaenus	*Rhynchaenus fagi*	1	124
				Sitona	Sitona	1	86
			Latridiidae	Cortinicara	Cortinicara	4	20,449
			Lucanidae	Dorcus	*Dorcus parallelipipedus*	1	81
			Nitidulidae	NA	Nitidulidae	4	2581
				Brassicogethes	Brassicogethes	3	19,328
			Phalacridae	Stilbus	Stilbus_testaceus	1	60
			Silphidae	Thanatophilus	Thanatophilus	1	21
			Staphylinidae	Tachyporus	*Tachyporus hypnorum*	1	38
		Dermaptera	Forficulidae	Forficula	Forficula	1	211
					*Forficula auricularia*	2	3728
		Diptera	Sciaridae	Scatopsciara	Scatopsciara	1	2275
			Stratiomyidae	NA	Stratiomyidae	1	94
		Ephemeroptera	Baetidae	NA	Baetidae	1	315
				Baetis	Baetis	1	1933
			Caenidae	Caenis	*Caenis horaria*	1	34
			Ephemerellidae	NA	Ephemerellidae	1	3260
				Serratella	Serratella	2	87,774
			Ephemeridae	Ephemera	*Ephemera vulgata*	1	46
		Hemiptera	Anthocoridae	Orius	Orius	1	84
			Aphididae	Brevicoryne	*Brevicoryne brassicae*	2	24
				Myzus	*Myzus persicae*	2	45
				Rhopalosiphum	*Rhopalosiphum padi*	1	64
			Miridae	NA	Miridae	2	206
				Lygus	Lygus	3	2934
					*Lygus pratensis/Lygus wagneri*	3	60,645
		Hymenoptera	Formicidae	Lasius	*Lasius niger*	1	10
			Pteromalidae	NA	Pteromalidae	4	239
		Thysanoptera	Thripidae	Thrips	*Thrips tabaci*	2	97

### 3.2 Diversity and Evenness Variation Within and Between Fields

Species accumulation curves derived from eDNA samples across the three management types showed similar patterns of taxa richness as the number of sampled sites increased (Figure 2b). Conventional and organic management exhibited the highest taxa richness, with the Lomolino model predicting maximum taxa richness values of 81.3 and 88.35, respectively. IP‐Suisse management showed lower taxa richness, with a predicted maximum of 76.4 taxa. Across all three management types, the Lomolino model provided the best fit, as indicated by the lowest AIC values. None of the accumulation curves approached an asymptote, suggesting that additional sampling would likely reveal further taxa.

**FIGURE 3:**
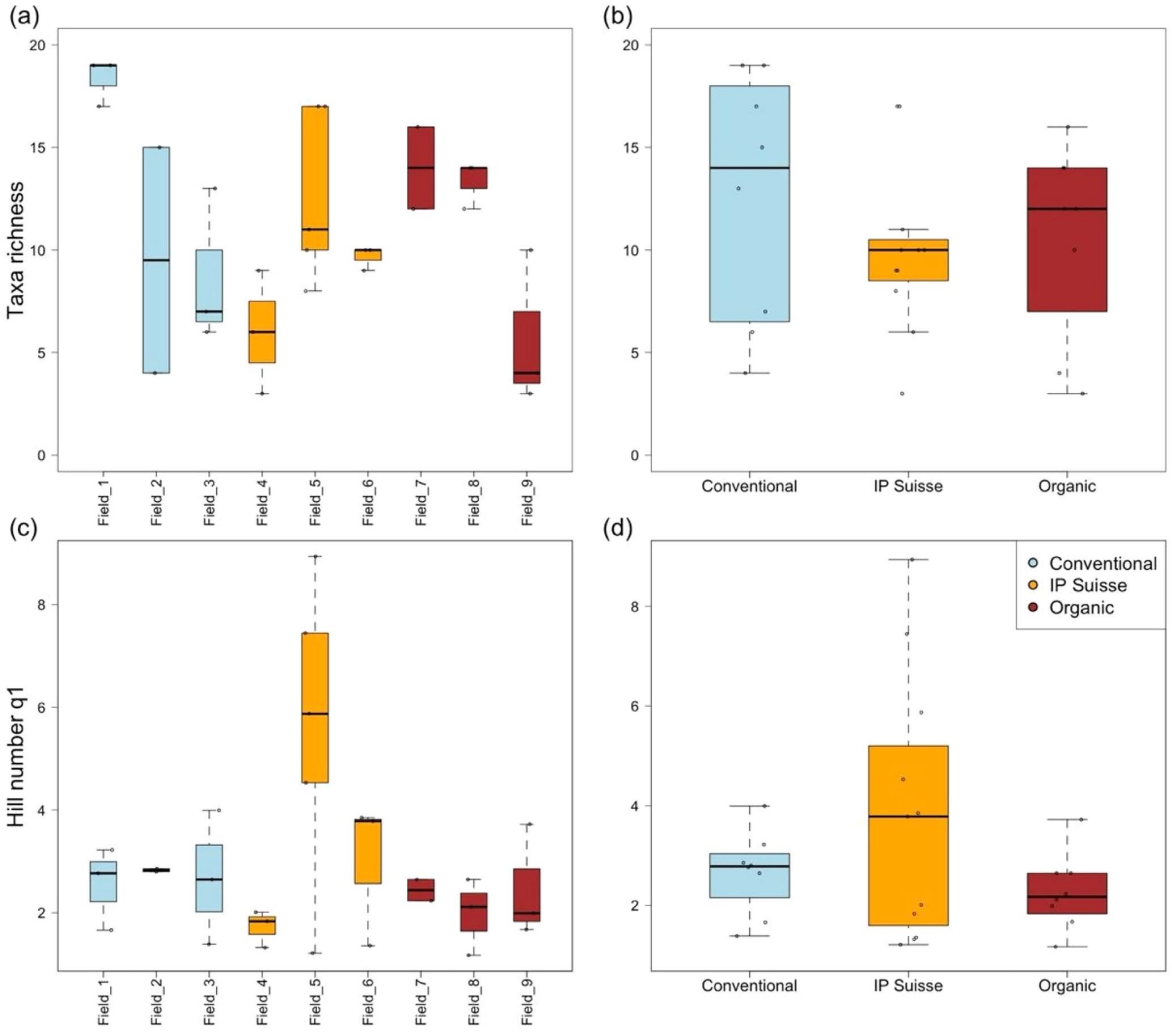
Numbers of taxa and evenness based on read counts recovered across management types and fields. The panels show: (a) a boxplot of taxa richness per sample as a function of Field, (b) a boxplot of taxa richness per sample as a function of management type (Conventional, IP‐Suisse, and Organic), (c) a boxplot of the q1 Hill number representing evenness per sample as a function of Field, and (d) a boxplot of evenness per sample as a function of management type.

The number of taxa detected per sample ranged from 3 to 19 taxa. We found that the variance in taxa richness between replicates within the same field (MS residual = 12.17) was significantly lower than the variance between samples of different fields (MS between = 49.35; *F* = 4.06, *p* = 0.007; Figure 3a). This result is strengthened by the reasonable assumption of equal variance across fields, with no evidence that within‐field variance differs among fields (Levene's test, *F* = 1.37, *p* = 0.27). We found that hexapod taxa richness did not vary significantly between management types (Tukey‐adjusted pairwise comparison, all *p* > 0.05). Similarly, a pairwise difference test between conventional compared to IP‐Suisse showed no difference (Tukey contrast; estimate = 1.27, 95% CI [0.89, 1.83], *p* = 0.26) and neither to organic (Tukey contrast; estimate = 1.13, 95% CI [0.78, 1.64], *p* = 0.74; Figure 3b). Also, no significant difference in hexapod taxa richness was found between IP‐Suisse and organic management types (Tukey contrast; estimate = 0.88, 95% CI [0.61, 1.28], *p* = 0.72).

Despite the mean square for variation in evenness among fields (5.54) being slightly higher than that within fields (2.65), the differences among fields were not statistically significant (ANOVA; *F* = 2.09, *p* = 0.092; Figure 3c). Therefore, we used a linear mixed‐effects model including Field as a random effect. The mixed model revealed that management types did not significantly influence evenness (Type III Wald *χ* ^2^ = 1.61, *p* = 0.448; Figure 3b). Overall, evenness varied more within fields than among fields but not significantly, and management type had no significant effect on evenness.

### 3.3 Composition Differences Among Fields

**FIGURE 4:**
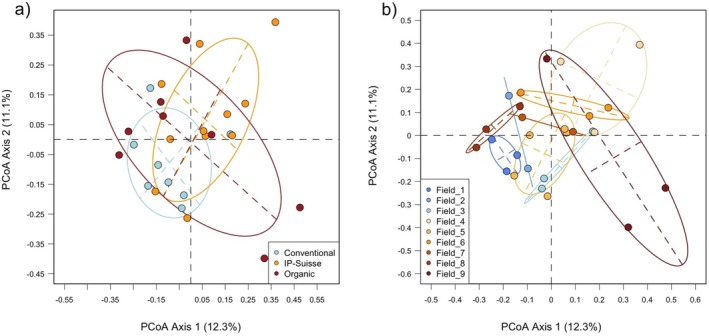
Ordination of the swabbing samples obtained from a PCoA analysis conducted on the community matrix. (a) PCoA based on Jaccard distances, colored by management type. (b) Same PCoA, colored by field type. Results indicate no major compositional differences between management types and that most of the sampled fields present the same species composition.

In terms of taxa composition, we found that the hexapod composition was not different among management types (Figure 4a,b). The mean Jaccard dissimilarity across all sites was high (βjac = 0.80 ± 0.1), indicating a strong difference in taxa composition mainly driven by taxa turnover (βjtu = 0.67 ± 0.2). The results of the PERMANOVA on βjac diversity indicate no effect of management types on hexapod community composition (*R* ^2^ = 0.09, *F* = 1.21, *p* = 0.12), with the model explaining only 19.2% of the variance. In contrast, we found a significant effect of sampling time (*R* ^2^ = 0.06, *F* = 1.53, *p* = 0.023) and no significant difference between fields (*R* ^2^ = 0.047, *F* = 1.27, *p* = 0.133). The PERMANOVA results on βjtu diversity also indicate that management types (*R* ^2^ = 0.07, *F* = 0.88, *p* = 0.62) and field (*R* ^2^ = 0.03, *F* = 0.66, *p* = 0.74) poorly explains the difference in hexapod β‐diversity variation while the time since the first sampling is marginally significant (*R* ^2^ = 0.075, *F* = 1.79, *p* = 0.075). Overall, 83% of the variation remains unexplained, suggesting that additional factors may influence β‐diversity. The Principal Coordinate Analysis (PCoA) of hexapod community composition based on Jaccard dissimilarity of presence/absence data shows that the first two axes explain 23.36% of the total variation (Axis 1: 12.29%, Axis 2: 11.07%). The substantial overlap between ellipses indicates considerable variation within each management type. The high dispersion of points within each management group further supports our finding that field‐specific factors explain the largest portion of variation in hexapod community structure.

## 4 Discussion

Environmental DNA technologies are increasingly recognized as transformative tools in biodiversity monitoring (Ruppert et al. 2019). Most eDNA studies to date have focused on aquatic systems due to the natural accumulation of DNA in water, which facilitates the detection of organisms across large spatial scales (Rees et al. 2014) or soil, but with a focus on below‐ground taxa for which it is more challenging to develop quantitative indicators (Salmi and Krzywoszynska 2025). The application of eDNA in agroecosystems remains comparatively underdeveloped and could be facilitated using vegetation surface sampling which can collect traces of macro‐organisms within well delimited areas (Allen et al. 2021). Our study demonstrates that drone‐assisted eDNA swabbing with metabarcoding analyses is an effective and scalable method for surveying hexapod diversity in agricultural fields, providing a noninvasive, repeatable, and efficient means of data collection. These findings are especially important in the context of agricultural sustainability. The transition toward more sustainable agricultural practices is often hindered by a lack of robust and scalable biodiversity monitoring tools that can demonstrate the cobenefits of, for example, reduced amounts of pesticides (Kremen and Miles 2012; Dainese et al. 2019). Traditional arthropod sampling methods, such as sweep‐netting or pitfall trapping, are invasive, labor‐intensive, time‐consuming, and difficult to implement consistently across large areas (Southwood and Henderson 2009). By contrast, drones can be preprogrammed to cover large fields systematically and can be integrated with automated swabbing tools, increasing spatial coverage while reducing labor demands.

We validated a method using drones to swab aerial plant surfaces in agricultural fields and showed that this approach consistently detects diversity of hexapod across multiple ecological guilds. By using this approach systematically across fields, we obtained a reproducible biodiversity signal at the field level. Within‐field replicates showed low variability compared to between‐field differences, indicating that our method reliably characterizes spatial biodiversity patterns. Independently of management types, fields with low diversity consistently showed species‐poor communities across replicates, while high‐diversity fields exhibited stable and rich hexapod communities. Considering evenness based on read counts, we detected no statistically significant differences within or between fields, and management type had no significant effect. These results suggest that the number of reads is roughly constant and their distribution similar across both management types and fields. This supports the use of aerial swabbing as a viable approach to quantify biodiversity in terrestrial systems is part of a rapidly growing body of work using vegetation and surface sampling to detect arthropod diversity (e.g., Thomsen and Sigsgaard 2019; Valentin et al. 2020; Banerjee et al. 2026), including swabbing of tree branches and forest canopies (Aucone et al. 2023; Kirchgeorg et al. 2024) and pathogen surveillance (Valentin et al. 2020). An increasing number of studies collectively suggest that swabbing in general is an efficient means to collect eDNA from a variety of taxonomic groups from invertebrates to vertebrates (e.g., Berard et al. 2025; Bodawatta et al. 2026; Guthrie et al. 2026). Vegetation swabbing, which can be aided by robots, therefore, represents a major new approach for the measurement of biodiversity, pests and pathogens in agricultural fields. While our method captured the diversity within fields, we did not detect a statistically significant effect of field management (e.g., organic vs. conventional treatment) on overall community composition. This result may either reflect the absence of effect on these treatments or limitations in our study design, including the relatively small number of sampled fields and the complex spatial structure of Swiss farmland and an incomplete sampling coverage, as none of the taxa accumulation curves reached an asymptote (Figure 2b), indicating that additional sampling would likely have revealed further taxa.

Management practices differ along more dimensions than those represented with the organic versus conventional categories, and more information on field‐level practices is required to assess their impacts on biodiversity. Moreover, Swiss agricultural landscapes are characterized by high habitat heterogeneity, with small field sizes and interspersed seminatural habitats (Herzog et al. 2005). These features likely increase spillover of mobile arthropods from surrounding areas into the field, or between fields, especially for taxa whose larval stages or overwintering sites are located in surrounding vegetation (Tscharntke et al. 2005). Landscape context is increasingly recognized as a dominant driver of arthropod diversity in agricultural systems (Chaplin‐Kramer et al. 2011). Therefore, biodiversity assessments based solely on in‐field observations may fail to account for ecological processes occurring at larger spatial scales. Although the area of seminatural habitat adjacent to each field was not recorded in this pilot study, we strongly recommend that future work incorporates landscape‐level metrics, including seminatural habitat area and connectivity indices as covariates, combined with detailed data on field practices and indicators of habitat availability and connectivity in the surrounding matrix (Le Provost et al. 2020). This would enable a more accurate evaluation of farm‐level contributions to biodiversity conservation.

Beyond these landscape effects, promoting biodiversity‐friendly farming practices also raises a broader question of trade‐offs with agricultural productivity, which is a key objective of agricultural sustainability policies. Organic farming and reduced pesticide use, for example, can benefit arthropod communities but may also lead to increased pest pressure and, in some cases, yield penalties (Seufert et al. 2012; Reganold and Wachter 2016). Similarly, the maintenance of seminatural habitats within or around fields can support beneficial insects and natural enemies, yet may reduce the total area available for crop production. These trade‐offs are context‐dependent and influenced by landscape configuration, crop type, and local management intensity. Our eDNA‐based monitoring approach, by enabling simultaneous tracking of pest and beneficial species at the field level, could help farmers and land managers better quantify these trade‐offs and identify management strategies that optimize both productivity and biodiversity outcomes.

In addition to measuring overall diversity, eDNA metabarcoding recovered a list of taxa informative for applied field management. For example, we detected high read counts for the rapeseed pest Ceutorhynchus pallidactylus (12,174 reads) and for Brassicogethes (Meligethes, pollen beetles), as well as low‐abundance reads for *Psylliodes chrysocephalus* , another rapeseed pest. These findings demonstrate the reliability of our sampling approach and its potential for pest surveillance. Early detection of pests using eDNA could allow for more timely and targeted management interventions, improving integrated pest management strategies (D'Ercole et al. 2022). Interestingly, we also detected forest‐associated pests such as the beech leaf miner (Rhynchaenus fagi) or orchard‐associated pests such as the green peach aphid ( *Myzus persicae* ), suggesting that eDNA sampling in agricultural settings can capture broader landscape‐level signals relevant to forest management. Moreover, we successfully identified beneficial taxa such as the seven‐spotted ladybird ( *Coccinella septempunctata* ) or the European earwig ( *Forficula auricularia* ), a well‐known predator of aphids in Brassica crops (Read et al. 1970). Such detections are particularly relevant for farmers aiming to transition away from synthetic pesticides toward nature‐based solutions. By identifying existing natural enemies in the field, eDNA tools can help optimize the use of biological control and support ecological intensification (Bommarco et al. 2013; Gurr et al. 2016). Importantly, the nondestructive nature of eDNA sampling means that it can be implemented repeatedly throughout the growing season to monitor the dynamics of pest and beneficial populations, their phenology, and spatial spread (Deiner et al. 2021).

Despite these promising results, several limitations remain. The resolution of taxonomic assignment depends heavily on the completeness and accuracy of DNA reference databases, which are still limited for many invertebrate taxa, particularly in understudied regions or for cryptic species (Andújar et al. 2018; Marquina et al. 2019). Here, we used primers for the 16S mitochondrial region because it was shown to outperform primers on the more commonly used CO1 region (Takenaka et al. 2023). However, reference databases for 16S are less developed than those for CO1 and thus the taxonomic assignment is less precise. Furthermore, while our protocol minimized contamination and standardized field protocols, eDNA signals can be influenced by various abiotic factors, such as UV radiation, precipitation, or wind, which may affect DNA degradation or dispersion (Barnes and Turner 2015; Harrison et al. 2019). An additional consideration specific to drone‐based swabbing is the possibility of contamination unrelated to in‐field biodiversity, arising from airborne eDNA being collected by the swab during flight. While our protocol did not include field‐flown negative controls to isolate this effect, the within‐field replicate consistency and the detection of crop‐stage‐specific pests (see above) suggest that direct surface contact, rather than airborne pickup, was the primary source of recovered eDNA. Future work should incorporate field‐flown negative controls to formally quantify the contribution of airborne eDNA to this sampling method. The source and persistence of eDNA signals in agricultural systems are not well known, and further studies are needed to optimize the sampling design. Future work should assess the temporal stability of eDNA signals across multiple time points and test sampling under different environmental conditions. Finally, benchmarking with traditional would be important to further validate the approach proposed here.

Our study introduces a novel, scalable approach for hexapod biodiversity monitoring in agricultural landscapes using drone‐based eDNA swabbing. The present study was designed as a pilot study aimed at demonstrating whether hexapod diversity could be reliably detected using drone‐assisted eDNA swabbing. Rather than providing an exhaustive biodiversity survey, our primary goal was to establish a proof of concept, with a particular focus on pest detection, which represents a key applied outcome for land managers and stakeholders. Future work will aim to broaden the taxonomic scope of this approach, including the exploration of additional insect primers as well as methods for mammal detection, such as the analysis of water collected around field margins. Nervertheless, this method offers a noninvasive, repeatable, and efficient alternative to traditional sampling, with the potential to generate robust, field‐level biodiversity data at unprecedented spatial scales. The successful detection of both pest and beneficial hexapod species underscores the applied value of this method for farmers and land managers. As eDNA reference databases improve and bioinformatic pipelines become more standardized, drone‐assisted eDNA metabarcoding is poised to become a core tool in the ecological monitoring toolbox. The broader integration of this approach with other sampling tools could significantly expand its scope and utility (Seeber et al. 2019). Ultimately, by enabling large‐scale, high‐resolution biodiversity monitoring, eDNA technologies can help bridge the gap between ecological research and agricultural policy, supporting evidence‐based strategies for sustainable land management worldwide.

## Author Contributions

**Camille Albouy:** conceptualization (equal), data curation (lead), formal analysis (lead), methodology (equal), project administration (equal), supervision (equal), visualization (equal), writing – original draft (equal), writing – review and editing (lead). **Louise Humbert:** data curation (equal), formal analysis (supporting), writing – original draft (supporting). **Kerstin Niedermeier:** resources (equal), writing – original draft (supporting). **Marc Chautem:** conceptualization (equal), funding acquisition (equal), project administration (lead), supervision (equal), writing – original draft (supporting). **Fabian Fopp:** resources (equal), visualization (equal), writing – review and editing (equal). **Christian Geckeler:** methodology (equal), writing – review and editing (equal). **Martina Luthi:** resources (equal), writing – review and editing (equal). **Sarah Thurnheer:** resources (equal), writing – review and editing (equal). **Virginie Marques:** data curation (equal), formal analysis (equal), writing – review and editing (equal). **Steffen Kirchgeorg:** methodology (equal), writing – review and editing (equal). **Ali Nasrallah:** writing – review and editing (equal). **Stefano Mintchev:** conceptualization (equal), writing – review and editing (equal). **Loïc Pellissier:** conceptualization (equal), funding acquisition (lead), project administration (lead), supervision (equal), visualization (equal), writing – original draft (lead).

## Conflicts of Interest

The authors declare no conflicts of interest.

## Data Availability

All the codes and data required to reproduce the analyses and figures are currently available at: https://figshare.com/s/b6f4bf91fcb261f23837.
